# Dissecting the genetic architecture of coronary artery disease by genome engineering

**DOI:** 10.1186/1753-6561-6-S6-P34

**Published:** 2012-10-01

**Authors:** David J Segal, Mital S Bhakta, Kumitaa Theva Das, Chongxiu Sun, Natalie M Grace, Jan A Nolta, Anne A Knowlton, David M Rocke, Scott I Simon

**Affiliations:** 1 Genome Center and Department of Molecular Medicine, University of California - Davis, Davis, CA 95616, USA; 2 Biomedical Engineering, University of California - Davis, Davis, CA 95616, USA; 3 Institute for Regenerative Cures, University of California - Davis, Davis, CA 95616, USA; 4 Cardiovascular Medicine, University of California - Davis, Davis, CA 95616, USA; 5 Division of Biostatistics, University of California - Davis, Davis, CA 95616, USA

## 

One of the greatest challenges facing biomedical research since the sequencing of the human genome has been to understand the role of genetic variation in human disease. Many genetic variants have been associated with common diseases. However, determining the functional consequences of these variants has been hard. Several variants are often inherited together in tightly linked blocks, making it difficult to determine the causative variant. People have millions of other genetic differences, making it difficult to correlate cellular phenotypes with a particular variant. Different gene sets are expressed in different cells, but it is difficult to extract disease-relevant cells from large numbers of patients. We describe a method with the potential to revolutionize the functional analysis of genetic variation, using custom nucleases to genetically modify individual variants in induced pluripotent stem cells. This process would provide unprecedented analytical power, present the first general method to determine if a variant is causative, and analyze function disease-relevant cell types. We will focus on variants at the 9p21 region of the genome that have been associated with coronary artery disease (CAD). The methods should provide a new way to unlock the wealth of data from genome-wide association studies, and to probe the genetic architecture of common diseases. We will describe our improved methods for inexpensive and rapid construction of highly active zinc finger and TALE nucleases to examine the functional role of polymorphisms at the 9p21 CAD risk locus.

